# A model for the biomass–density dynamics of seagrasses developed and calibrated on global data

**DOI:** 10.1186/s12898-019-0221-4

**Published:** 2019-01-25

**Authors:** Vasco M. N. C. S. Vieira, Inês E. Lopes, Joel C. Creed

**Affiliations:** 1 MARETEC, Instituto Superior Técnico, Universidade Técnica de Lisboa, Av. Rovisco Pais, 1049-001 Lisbon, Portugal; 2 Departamento de Ecologia, Instituto de Biologia Roberto Alcântara Gomes, Universidade do Estado do Rio de Janeiro, Rua São Francisco Xavier 524, Rio de Janeiro, RJ 20559-900 Brazil

**Keywords:** Above-ground biomass, Cymodoceae, Halodule, Interspecific boundary line, Logistic growth, Thalassia, Zostera

## Abstract

**Background:**

Seagrasses are foundation species in estuarine and lagoon systems, providing a wide array of services for the ecosystem and the human population. Understanding the dynamics of their stands is essential in order to better assess natural and anthropogenic impacts. It is usually considered that healthy seagrasses aim to maximize their stand biomass (g DW m^−2^) which may be constrained by resource availability i.e., the local environment sets a carrying capacity. Recently, this paradigm has been tested and reassessed, and it is believed that seagrasses actually maximize their efficiency of space occupation—i.e., aim to reach an interspecific boundary line (IBL)—as quick as possible. This requires that they simultaneously grow in biomass and iterate new shoots to increase density. However, this strategy depresses their biomass potential.

**Results:**

to comply with this new paradigm, we developed a seagrass growth model that updates the carrying capacities for biomass and shoot density from the seagrass IBL at each time step. The use of a joint biomass and density growth rates enabled parameter estimation with twice the sample sizes and made the model less sensitive to episodic error in either of the variables. The use of instantaneous growth rates enabled the model to be calibrated with data sampled at widely different time intervals. We used data from 24 studies of six seagrass species scattered worldwide. The forecasted allometric biomass–density growth trajectories fit these observations well. Maximum growth and decay rates were found consistently for each species. The growth rates varied seasonally, matching previous observations.

**Conclusions:**

State-of-art models predicting both biomass and shoot density in seagrass have not previously incorporated our observation across many seagrass species that dynamics depend on current state relative to IBL. Our model better simulates the biomass–density dynamics of seagrass stands while shedding light on its intricacies. However, it is only valid for established patches where dynamics involve space-filling, not for colonization of new areas.

**Electronic supplementary material:**

The online version of this article (10.1186/s12898-019-0221-4) contains supplementary material, which is available to authorized users.

## Introduction

Seagrasses are dominant primary producers in coastal systems, and particularly in estuarine and lagoon ecosystems. Worldwide, seagrasses provide a wide array of ecosystem services that vary substantially with geographical location and the morphological and demographic characteristics of the species [1]. By inhabiting the coastline, seagrasses are subject to negative terrestrial human mediated impacts. The most frequent is eutrophication, which affects seagrasses directly through the deleterious effect of pollutants and indirectly by promoting blooms of opportunistic and epiphytic algae that may shade and smother seagrass stands [2–6]. Decreases in biomass, shoot density and growth rates are common consequences [7–11].

Seagrasses have a modular construction. Buried in the sediment, the rhizomes elongate and laterally grow new nodes with shoots. The internode length depends on the species and on its growth mode and clonal-growth plasticity, as was demonstrated in the reanalysis by Vieira et al. [12] of the Dadae Bay case study [13]. Multiple shoots can occur on a single rhizome, with their appearance restricted to rhizome nodes. Although species specific, the clonal growth of seagrass stands usually takes two stages [14]: during the earlier years of patch formation the stands elongate their rhizomes in a Diffusion-Limited Aggregation model to occupy the available substrate. Once the patch is established, it changes to an Eden strategy aiming at spreading to the neighbouring areas. Within the saturated patch, new space only becomes available upon the death of old shoots. Renton et al. [15] explicitly modelled the survival and growth of rhizomes and shoots to optimize transplant strategies for restoration. A different approach has been preferred when modelling established stands to quantify their primary production and total biomass. Plus et al. [16] estimated shoot density, above-ground biomass and below-ground biomass using a set of differential equations with a linear structure that ignored the environmental carrying capacity. Irrespective of the stand’s developmental stage and modelling approach, the environmental factors most commonly influencing seagrass growth rate are temperature, irradiance and concentration of inorganic nutrients [8, 14–18].

**Fig. 1:**
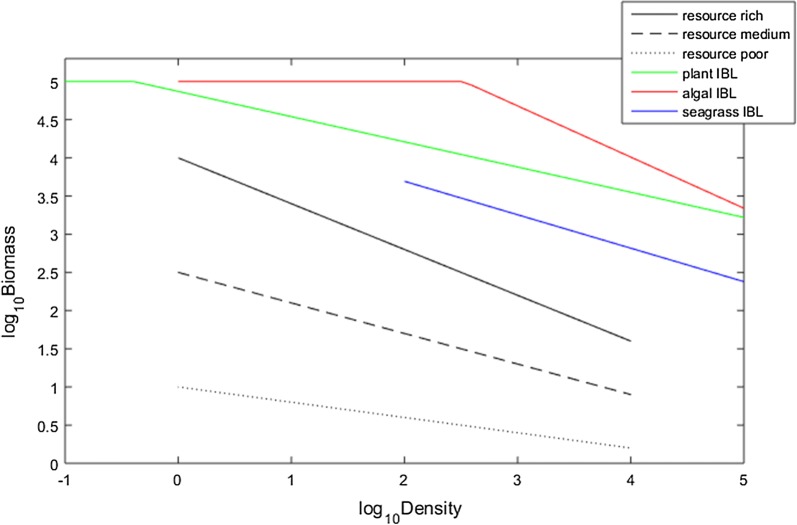
Biomass–density relations. Theoretical schematic of self-thinning under different resource levels and observed interspecific boundary line (IBL) of algae, terrestrial plants and seagrasses

Biomass–density relations that may relate to yield became central to plant demography in the 1950’s [19–21]. Significant insights into the dynamics of plant stands can be inferred from bi-logarithmic plots with log_10_D in the x axis and log_10_B in the y axis, where D is density in numbers of individuals (ramets) per unit area (ind m^−2^) and B is stand biomass per unit area (g DW m^−2^). The time trajectory of a monospecific even-aged stand under crowded conditions is named the “intraspecific dynamic biomass–density relation”, or alternatively the “self-thinning line”. While the stands endure active growth, crowding induces mortality of the weaker, which in turn opens space for the growth of the fitter. This iterative process generates a line with negative slope reflecting the environmental carrying capacity and degree of intraspecific competition [20, 21] (Fig. 1). Above any self-thinning line is placed a boundary line that no stand or species can pass and reflects the maximum possible efficiency of space occupation [12, 20–22] (Fig. 1). This boundary line is termed the Interspecific Boundary Line (IBL) and is given by log_10_B = β_0_ + β_1_∙log_10_D. The IBL for terrestrial plants has coefficients β_0_ = 4.87 and β_1_ = − 0.33 [21]. Recently, algae were demonstrated to occupy space more efficiently than plants [22], with the algae IBL exhibiting coefficients β_0_ = 6.69 and β_1_ = − 0.67 placed above the plant IBL (Fig. 1). Nevertheless, there was a threshold of log_10_B ≈ 5 that neither algae nor plants were able to cross [22]. The perpendicular distance from each algal stand to their boundary reflected its specific efficiency of space occupation and was used to discriminate among taxa, functional groups, clonality or latitude [22]. Soon after, Vieira et al. [12] demonstrated that seagrasses are also limited by their own IBL. With coefficients β_0_ = 4.569 and β_1_ = − 0.438, the seagrass IBL was placed far below the algae and plant boundaries (Fig. 1).

**Fig. 2:**
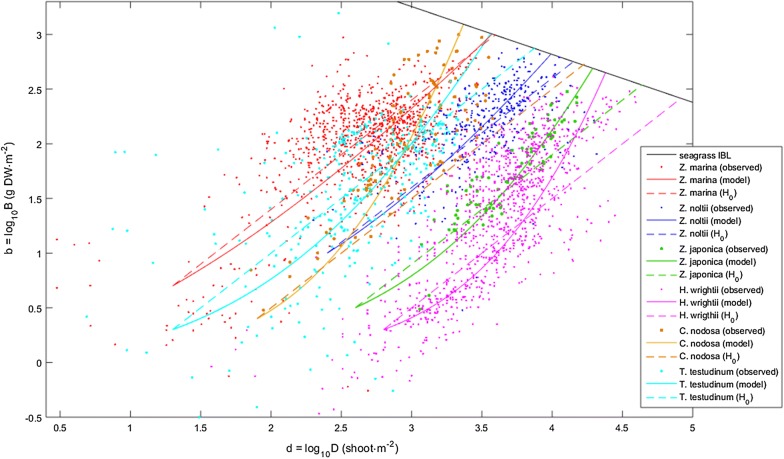
Biomass–density relations of seagrasses. Observed (obs) and estimated by the allometric instantaneous growth model (model) or the isometric null hypothesis (H_0_)

Self-thinning does not apply to many clonal algae and plants because modules (ramets) are physically interconnected allowing the sharing of acquired resources and offsetting competition [23–29]. Although not necessarily self-thinning, seagrasses [12], terrestrial clonal plants [23] and clonal algae [22] have been demonstrated to be limited by their respective IBL, as are non-clonal macrophytes. Therefore, it is both possible and legitimate to use their stands’ distances to their IBL as estimators of their efficiencies of space occupation. When doing such estimation, Vieira et al. [12] found that seagrasses tend to develop biomass and shoot density in a trajectory approximately perpendicular to their IBL. Hence, when the environment is favourable, seagrass stands grow approaching their IBL by simultaneously increasing shoot density and stand biomass (Fig. 2). On the other hand, when the environment is unfavourable, seagrass stands shrink back and depart their IBL by simultaneously decreasing their shoot density and stand biomass (Fig. 2). This particular temporal biomass–density scenario suggests that seagrasses (i) grow to maximize the efficiency of space occupation and not just biomass, and (ii) aim at the quickest route to maximize this efficiency.

In this study we developed a model for the growth of established seagrass stands that mimicked the observed patterns mentioned above. It required that the growth model solved simultaneously for biomass and density considering one carrying capacity for each of these properties. To develop such a model, we nested logistic functions for the stand biomass and shoot density with the carrying capacities iteratively updated by selecting the IBL coordinates closest to the current stand coordinates. This represents a new paradigm in modelling seagrass meadows as former models ignored (i) the coordinated biomass and shoot density growth, (ii) the existence of carrying capacities for biomass and shoot density, and (iii) their dependency on the efficiency of space occupation. We calibrated a model for each of the six studied species. Their simulations were analysed regarding their ecological implications as well as comparisons among species.

## Methods

Vieira et al. [12] gathered data comprising the biomass and shoot density presented in 32 studies of ten seagrass species distributed worldwide. The *Halodule wrightii* data was provided by Dr. Joel Creed and Dr. Kenneth Dunton. The data from Plus et al. [17] was provided by Dr. Martin Plus. The remaining data were retrieved from the respective publications. The compilation of data, carried out during the years 2017 and 2018, used the Google search engine as well as the search engines in the webpages of all cited publications, and included the keywords ‘biomass’, ‘density’, ‘seagrass’ and the species scientific denominations. Vieira et al. [12] also searched the publication listings of the most cited authors in the subject and the reference lists of the cited works. This data was provided as Additional file 1 associated to that publication. Here, we used a sub-set of this data comprising the biomass and shoot density presented in 24 studies of six seagrass species. These were the species for which the existence of time series data allowed the determination of growth rates fundamental for this modelling. All data used are included in Fig. 2. The software estimating the parameters and running the model are provided as Additional file 1.

### The biomass–density instantaneous growth model

Following the bulk literature on biomass–density relations, the biomass (B in g DW m^−2^) and density (D in shoots m^−2^) were replaced by b = log_10_B and d = log_10_D. Coincidently, these correspond to instantaneous rates (although traditionally use the e base), allowing the application of linear algebra to non-linear processes, and thus standardizing per day (i.e., ∆b/∆t and ∆d/∆t) growth rates that in their original studies related to quite different time intervals. This advantage of instantaneous over finite rates has made them the most suited for studies in fisheries [30, 31] and evolutionary [32, 33] ecology.

Depending on the environmental conditions, the stands approached or departed the seagrass IBL along a path roughly corresponding to the central tendency observed for each species (Fig. 2). This was estimated by Principal Components Analysis (PCA) based on the biomass–density covariance matrix. PCA is a Type II regression, a class of methods (also including reduced major axis—RMA) that has been demonstrated to be better suited for data without a hierarchical structure and/or with approximate x and y variances [34–36], as is the case of biomass–density relations [20, 37, 38]. PCA and RMA tend to be complementary, with one excelling where the other fails. However, when applied to biomass–density data, PCA often performs better than RMA [39–41]. With these seagrass data, both methods were generally equally good, and RMA performed conspicuously less well only when applied to *Cymodocea nodosa* (Ucria) Ascherson (1870). Having decided to use PCA, the central tendency was given by the dominant principal component—i.e., the one with the larger eigenvalue. Its slope (i.e., α_1_ = ∆b/∆d) was taken from its eigenvector, with the b loading corresponding to ∆b and the d loading corresponding to ∆d. The angle θ between the central tendency and the d horizontal axis was estimated from the slope i.e., θ = arctg((∆b/∆d). This angle weights the allometry in the biomass growth relative to the density growth. Larger θ implies more biomass grown per unit increase in shoot density.

The θ under the null hypothesis (H_0_) of isometric biomass–density growth was estimated for comparison. In this case the increase in the stand’s biomass per area is exclusively a consequence of iteration of new shoots without any increase in individual biomass. Obviously, shoots are not “born” at adult size, so we assume that the time frame for growth to adult size is rapid relative to new shoot production. This isometric biomass–density growth represents a situation where a cohort of shoots reaches a fixed adult size before the emergence of the next cohort. Because the axes of the biomass–density plot are in logarithmic scales, the slope of the b:d central tendency (α_1_) observed for each species represents the exponent in their allometric relation $B = 10^{{\alpha_{0} }} D^{{\alpha_{1} }}$. Under the isometric null hypothesis this exponent is 1, leading to θ = 0.785. Consequently, irrespective of the species, θ > 0.785 implied an allometric biomass–density growth due to older shoots keeping increasing their biomass.

**Fig. 3:**
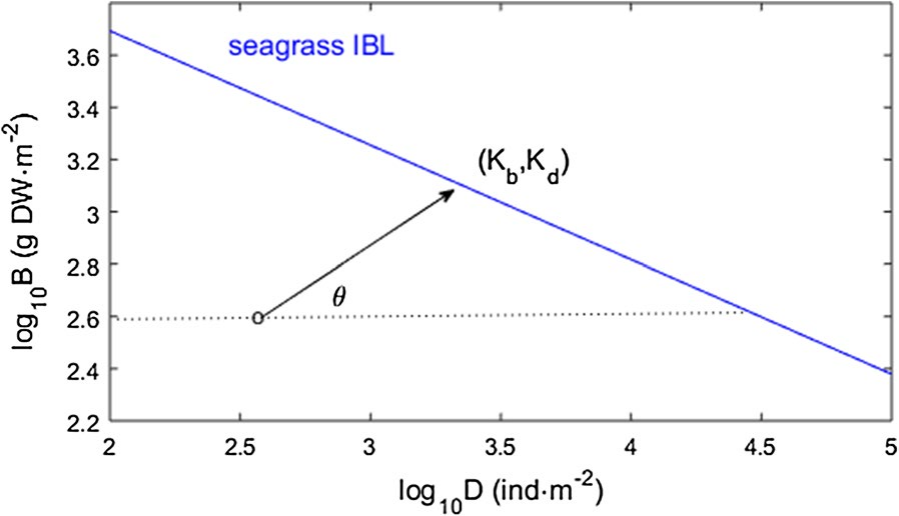
Iterative update of the biomass and density carrying capacities

The carrying capacities K_b_ and K_d_ were taken from the IBL (Fig. 3) in two situations: (i) at each iteration of the instantaneous growth model, and (ii) during model calibration, for the estimation of the growth parameter r. Thus, K_b_ and K_d_ corresponded to the intersection of the IBL with a straight line passing by the stand’s location during the iteration and preserving the slope (and thus, also the θ) previously estimated from the central tendency:

1 $$K_{d} = \frac{{b - \beta_{0} - \alpha_{1} d}}{{\beta_{1} - \alpha_{1} }}$$

2 $$K_{b} = \beta_{0} + \beta_{1} K_{d}$$

In the core of the biomass (B) and shoot-density (D) growth models were exponential growth functions where the B and D one time step ahead were given by B_t+1_=R_B_∙B_t_ and D_t+1_ = R_D_∙D_t_. Consequently, the interval growth rates corresponded to R_B_ = B_t+1_/B_t_ and R_D_ = D_t+1_/D_t_. Changing units to b and d led to R_B_ = 10^∆b^ and R_D_ = 10^∆d^, with ∆b = b_t+1_ − b_t_ and ∆d = d_t+1_ − d_t_. This adaptation enabled the application of the logistic growth function to instantaneous growth rates preserving its typical sigmoidal-shaped curve (Eqs. 3 and 4). The θ estimated for each species described the proportionality between its biomass and density growth rates, allowing the model to include a single general growth rate (r) i.e., the biomass specific r_b_ = r∙sinθ while the density specific r_d_ = r∙cosθ.

3 $$\frac{\Delta b}{\Delta t} = r \cdot b\left( {\frac{{K_{b} - b}}{{K_{b} }}} \right)\sin \theta$$

4 $$\frac{\Delta d}{\Delta t} = r \cdot d\left( {\frac{{K_{d} - d}}{{K_{d} }}} \right)\cos \theta$$

During model calibration, the instantaneous logistic growth functions were linearized (Eqs. 5 and 6). Scaling b and d to their estimated carrying capacities yielded the dimensionless quantities b/K_b_ and d/K_d_, most often ranging from 0 and 1 although small negative values also occurred from very small biomasses and/or densities. K_b_ and K_d_ were previously estimated from Eqs. (1) and (2). The solution in Eqs. (5) and (6) with both the horizontal (x) and vertical (y) axis in units of day^−1^ allowed the merging of biomass and density data into a single estimation of r, increasing its accuracy. In this case, r is both the slope and the intercept of the regression line.

5 $$\frac{\Delta b}{\Delta t} \cdot \frac{1}{b \cdot \sin \theta } = r\left( {\frac{{K_{b} - b}}{{K_{b} }}} \right) = r - r\frac{b}{{K_{b} }}$$

6 $$\frac{\Delta d}{\Delta t} \cdot \frac{1}{d \cdot \cos \theta } = r\left( {\frac{{K_{d} - d}}{{K_{d} }}} \right) = r - r\frac{d}{{K_{d} }}$$

This model structure has, apparently, four parameters: the species-specific biomass–density central tendency (θ), the biomass–density joint growth rate (r), and the biomass and density carrying capacities, respectively K_b_ and K_d_. However, K_b_ and K_d_ are not true parameters, rather being iterated from the seagrass IBL, a “universal” boundary line common for all seagrass species. Thus, its β_0_ and β_1_ coefficients are universal constants and not parameters to be calibrated. At present these constants were estimated from data of only 10 species [12], but hopefully future studies will provide a more comprehensive dataset to establish the better placement of this IBL and the value of its coefficients.

We tested the advantage of our model by comparing with the state-of-the-art in modelling the dynamics of seagrass meadows. This was the MEZO-1D with explicit independent parameterization of shoot density and above-ground biomass, and applied to *Z. noltii* in the Thau Lagoon [16]. Then, we ran our model in operational mode using this same *Z. noltii* data. The K_d_, K_b_ and growth rate (r) were estimated for each time interval from the observed biomass and density using Eqs. (1, 2, 5 and 6). The biomass and density were forecasted for the next time step i.e., b_t+1_=b_t_ + Δb and d_t+1_=d_t_ + Δd. The b_t_ and d_t_ were the observed biomass and density while the Δb and Δd were estimated solving Eqs. 3 and 4 for Δb and Δd.

## Results

Excepting *Z. marina*, the biomass–density growth of all other tested seagrasses was largely allometric (Table 1), meaning that biomass increased both from the emergence of new shoots and the growth of old shoots. In *Z. marina*, the biomass–density growth was almost isometric. These results were independent of the time interval between consecutive samples. The median interval for *Z. marina*, *Z. japonica* and *C. nodosa* was roughly 1 month. For the *Z. noltii*, *H. wrightii* and *T. testudinum* the bulk of the intervals varied among 2, 3 and 4 months. The biomass–density growth trajectories simulated by allometric (instantaneous growth) and isometric (null hypothesis) models were generally largely different (Fig. 2). These differences depended on how far the starting point was from the seagrass IBL. With *Z. marina*, the starting point needed to be far below the IBL for the allometric (instantaneous growth model) and isometric (null hypothesis) models to yield conspicuously different trajectories. Otherwise, their trajectories were very similar. By disregarding the biomass growth of older shoots, the simulations of isometric biomass–density growth reached the seagrass IBL (i.e., the carrying capacity) over-estimating densities while under-estimating biomasses. The variation of θ among species was relatively narrow (Table 1). Nevertheless, the smaller θ (*Z. marina*), the median θ (*T. testudinum*) and the larger θ (*C. nodosa*) were found among the species with larger shoots reared at smaller densities, demonstrating that the allometry in the biomass–density growth was independent of species morphotypes.

**Table 1:** Instantaneous growth model parameters

Species	H_0_	Instantaneous growth model		
	Θ	θ	r (max)	r (min)
*C. nodosa*	0.785	1.052	0.029	− 0.03
*H. wrightii*	0.785	1.04	0.055	− 0.075
*T. testudinum*	0.785	0.948	0.065	− 0.065
*Z. japonica*	0.785	0.966	0.04	− 0.055
*Z. marina*	0.785	0.836	0.043	− 0.043
*Z. noltii*	0.785	0.874	0.03	− 0.025

Higher θ mean larger responses of biomass relative to density. The null hypothesis (H_0_) is the isometric biomass–density growth

**Fig. 4:**
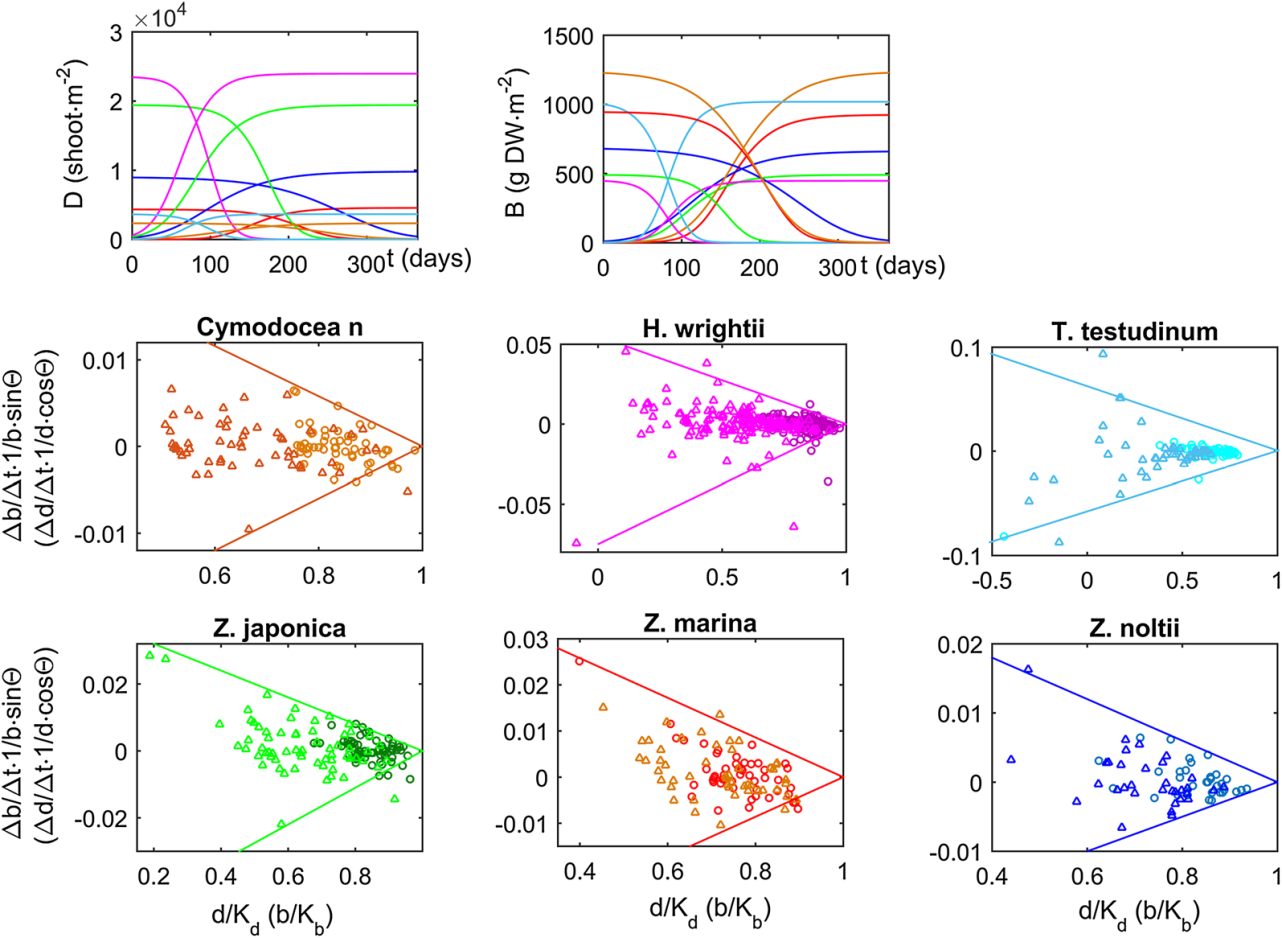
Model calibration. Inferred for six seagrass species using data retrieved from stands worldwide. Data relative to biomass (triangle) or density (circle)

**Fig. 5:**
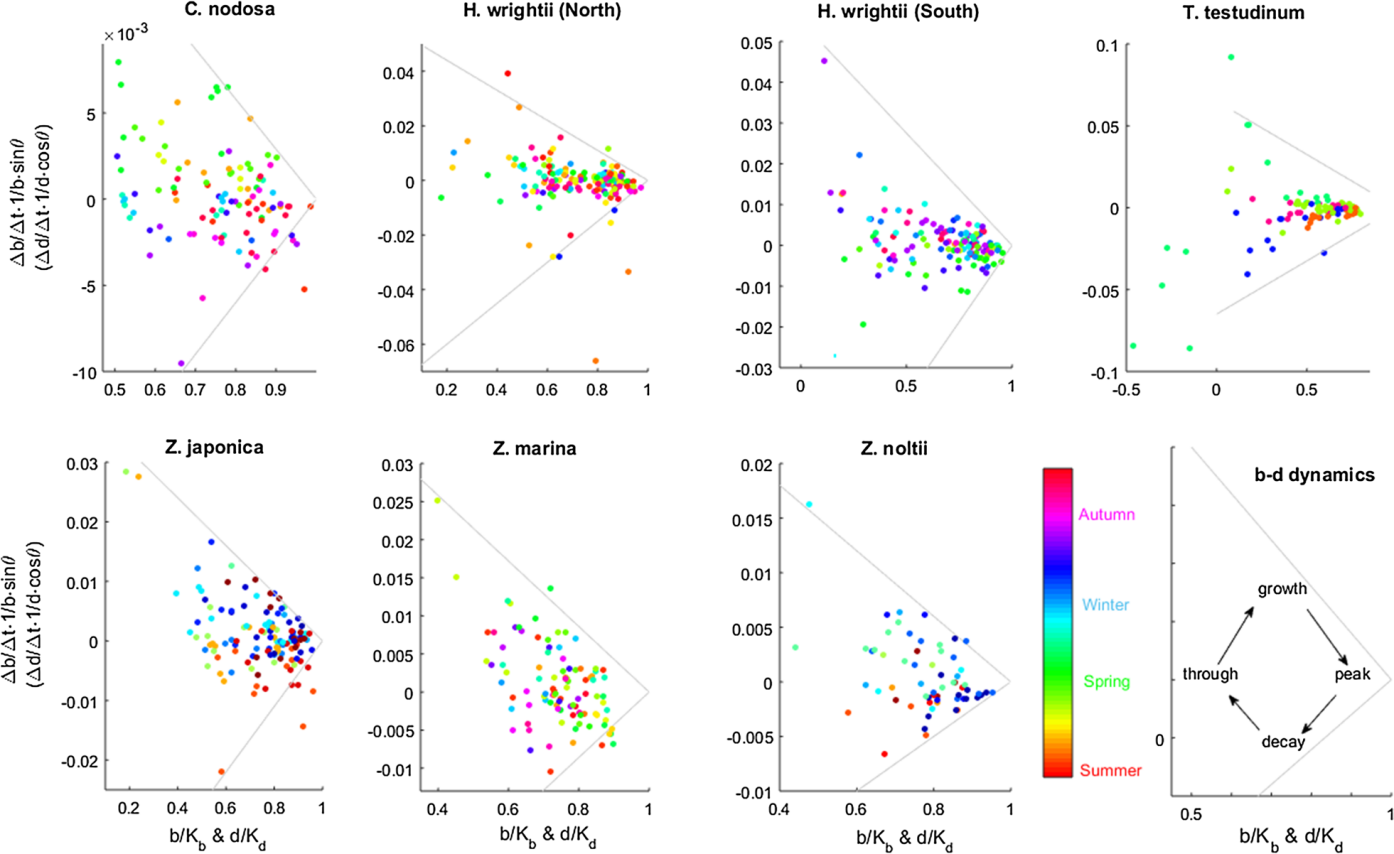
Growth seasonality. Inferred for six seagrass species using data retrieved from stands worldwide

The calibration of the instantaneous growth model showed that each species is systematically bounded within a minimum (decay) and a maximum (growth) rate, beyond which observations are scarce (Table 1 and Fig. 4). The estimated maximum rates report the best performance of each species observed on a regular basis, enabling most species to attain the seagrass IBL—i.e., to reach their carrying capacities—in just a few months (Fig. 4). These maxima occur consistently (i.e., fit the same line) along the full range of observed stand biomasses and densities (i.e., along the b/K_b_ and d/K_d_ axis), thus corroborating the adequacy of this modelling approach in describing stand dynamics. Comparing among species, the maximum rates were unrelated to shoot size and shoot density. Both larger and smaller maxima were found among the species with larger shoots reared at smaller densities (Table 1). The estimated decay rates occurred consistently along the full range of observed stand biomasses and densities, represented the worst performance of each species observed on a regular basis, and resulted in most species shrinking far away from the seagrass IBL in just a few months (Fig. 4). The maximum decay rates were also unrelated to morphometry. Both larger and smaller maxima were found among the species with larger shoots reared at smaller densities (Table 1). Maximum growth and decay rates were of similar magnitudes. Nevertheless, the episodic occurrence of faster decay rates should relate to adverse extreme events. Bounded within the maximum growth and decay rates, for some species (particularly for *C. nodosa* and *T. testudinum*) it was easy to identify a seasonal dynamic cycling through growth, peak, decay and trough of the stands’ biomass–density relation (Fig. 5). For other species the seasonal pattern may be blurred by the spatial variability.

The resulting instantaneous growth models simulated well the dynamics of the six tested seagrass species. All trajectories forecasted in the biomass–density plot fit the central tendencies of their respective species (Fig. 2). Nevertheless, it is unclear whether the sensitivity of the trajectories to the initial conditions matched reality or constituted a model weakness. On the one hand, the observation of a clear oblique pattern towards the IBL suggests some sort of control mechanism with negative feedback keeping some species in their respective biomass–density narrow bands. On the other hand, the scatter around the central tendencies, particularly large in some species, casts doubt on the existence or on the efficacy of such a control mechanism.

**Fig. 6:**
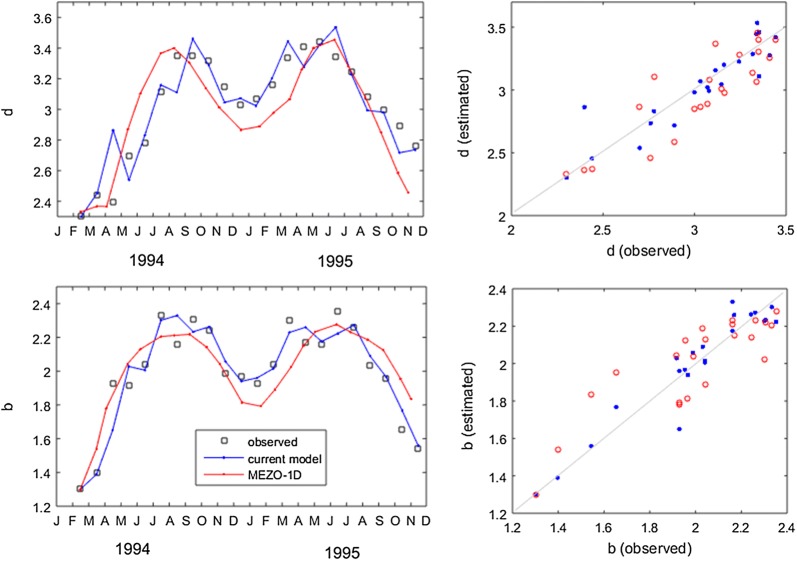
Seagrass demographic models. Left panels have the b and d time series yield by our model run in operational mode and of MEZO-1D in long range forecast. Right panels have model validation

Both models captured the seasonal variation observed in *Z. noltii* in the Thau Lagoon: b and d both varied by 1 unit, and predictions were generally within 0.2 units (Fig. 6). This seasonal variation was achieved in MEZO-1D through a time-varying carrying capacity set indirectly through resource availability, hence producing smooth seasonal variation. Our model, instead, generated dynamic increases and declines in seagrass through time-varying r (i.e., negative in fall and winter, positive in spring and summer), while K_d_ and K_b_ varied in time but always along the IBL. Part of our model fit was derived from implementation in operational mode, thus preventing error propagation and amplification through time; MEZO-1D applied in operational mode (always updated from observed rather than predicted values) would likely also fit better. Nevertheless, our model correctly tracked the contributions of shoot loss and smaller size at the end of the time series, whereas MEZO-1D overestimated biomass per area and underestimated shoot density.

## Discussion

Our model and findings are only valid for established stands (or patches), where the below ground system is already spread through the whole surface and the occupation of the space available above-ground is only a consequence of the shoot dynamics (their growth and mortality). This limitation is a consequence of our model being based on the biomass–density relation and Interspecific Boundary Line estimated from (and for) established stands. These fundamental ecological principles applied to terrestrial plants [19–21] and algae [22] are also only valid for established stands, and in the generality of the plant and algae cases the colonization of the free surface is by the dispersal of seedlings or sporelings instead of rhizome elongation. Seagrass stands that are not fully established require long term expansion of their rhizomes to fully occupy the free surfaces [14]. Consequently, seagrass populations are unstable when their below-ground system is harmed or destroyed. Even damaging their clonal integrity or just severing the apical meristem can be enough to significantly reduce their production of shoots, leafs and biomass [42]. Contrasting with the large timeframe required for the establishment of new stands (or patches), our results demonstrate that, when their below ground system is established and healthy, seagrasses quickly attain their maximum efficiency of space occupation. Even starting from poor conditions (i.e., low biomass and/or low density), the simultaneous increase in shoot density and biomass gets seagrasses up to their maximum efficiency in a few months. Contributing for such a quick response may be the occurrence of dormant shoots ready to develop upon the physiological perception of favourable environments in those species that have them [43] and clonal shoot production rates that are inversely related to shoot density in other species [44]. We therefore conclude that: (i) the health of the stand’s below-ground system is a key aspect for the stability of seagrass stands, and (ii) fast vs. slow growing species only makes sense when addressing the below-ground growth. But once this is established, all species can grow their shoot density and above-ground biomass to their carrying capacities in just a few months.

Our results confirm that postulated by Vieira et al. [12] that seagrasses are programmed to maximizing their efficiency of space occupation (i.e., approach their seagrass IBL) as quickly as possible by simultaneously adjusting biomass and shoot density. For accurate simulations of this adjustment the correct allometric biomass–density growth algorithm is fundamental. Disregarding the simultaneous adjustment of biomass and shoot density, or using the wrong allometric relation, inevitably leads to extremely biased estimates of biomass, density and their carrying capacities. Distinct seagrass species show different patterns in their simultaneous growth in biomass and in shoot density. *Z. marina* was the only species whose stands showed almost isometric growth, implying that the addition of biomass resulted mainly from the emergence of new shoots. In all other tested species the biomass–density growth was allometric, implying that the addition of biomass resulted both from the emergence of new shoots and the continuous growth (or weight increment) of old shoots. Our model contributed with developments that are fundamental for the modelling of this biomass and shoot density dynamics of seagrasses. The MEZO-1D [16] is, to our knowledge, the state-of-the-art in modelling seagrass stands. Yet, it disregards (i) the coordinated growth of biomass and shoot density, (ii) the existence of carrying capacities for these two properties, and (iii) the carrying capacities being set by the efficiency of space occupation.

Ideally, seagrass demographic models like the MEZO-1D should be merged with ours, and here we put forward a way to do it: the general framework of our model must be preserved as the ultimate carrying capacity is set by the IBL (the maximum possible efficiency of space occupation) and not by nutrients or light. Nevertheless, these other factors do set a secondary carrying capacity, enabling the stands to approximate the IBL (r > 0) or leading them to depart from it (r < 0). The most obvious solution is setting a secondary carrying capacity for biomass (b_K_), then evaluate the stands placement relative to it (b) and scale the growth rate to this differential i.e., r∝b_K_ − b. This way, a stand grows towards the IBL while it is not being limited by resources (i.e., r = λ(b_K_ − b) > 0, with λ a positive scaling constant) but departs the IBL when it is being limited by resources (i.e., r = λ(b_K_ − b) < 0). This simple solution also postulates that stands with smaller shoots summing up to lower stand biomass are less constrained by resources. This dynamic is supported by the results presented in Fig. 5, where positive and negative r often changed seasonally. So, despite the advances brought about by our model, much improvement is still possible and required. Better quality data, particularly with finer temporal resolution, should allow better calibration and assessment of the seasonal dynamics. Another fundamental aspect for the development of the current model is its sensitivity to initial conditions. It is uncertain whether this represents reality or is a mathematical flaw. The large scatter around the central tendency of each species biomass–density plot suggests that at least part of this sensitivity is real.

It is also reasonable to expect that the different biomass–density strategies reflect the different morphological and physiological limits of each species. The presence of dormant shoots ready to develop upon the physiological perception of favourable environments in *Thalassia testudinum* [43] may be one specific differential with a strong influence on the balance between the coordinated growth in biomass and shoot density. The existence of dormant shoots has also been suggested though not confirmed in *Cymodocea nodosa* [43]; in this study this species was observed to have the strongest biomass dominance in the biomass–density coordinated growth (see Table 1), implying that its seasonal variation in biomass greatly surpassed its seasonal variation in density. It might be that “waking-up” dormant shoots and getting them to resume growth of leaves could explain the efficiency of space occupation by *Cymodocea nodosa*. This may also help explain the narrow biomass–density band occupied by *Cymodocea nodosa*, as was observed both in this study and by Vieira et al. [12]. For the calibration of the current model it is fundamental to know whether the counts of shoot-density in the data included (or not) dormant shoots. For the development of more comprehensive mechanistic models the presence of dormant shoots should be considered.

Vieira et al. [12] demonstrated that seagrasses generally followed the same seasonal pattern, with the spring and summer corresponding to the favourable season and the autumn and winter corresponding to the unfavourable season, but still a differentiation occurred among seagrasses in their maximum efficiency of space occupation. Together, our study and the study by Vieira et al. [12] unveiled further details about the biomass–density dynamics of seagrasses. By the end of the favourable season the seagrass stands may already be at (or close to) their carrying capacities set by their seagrass IBL, while unbounded by any light or nutrient availability. In these cases the stands do not grow further simply because it is physically impossible for them to occupy more space, and not because the environmental conditions are less adequate. On the other hand, our results showed that seagrasses require some months to attain their IBL, and some species require more time than others. The question is raised as to whether the lower maximum efficiencies observed in some species by Vieira et al. [12] are a direct limitation of their morphology or result from growth rates that were too slow for the short favourable season. The latter case may explain our results, and those by Vieira et al. [12], demonstrating that *Z. marina* and *T. testudinum* have a potential efficiency of space occupation better than that reported so far. It may be that in the studied sites, following the harsher winters, the favourable summers did not last long enough for the stands to grow to their maximum.

## Conclusions

Our model, built on the new paradigm about the joint biomass–density dynamics of seagrasses, sheds light on the intricacy of their ecology. Consequently, it simulates the dynamics of seagrass stands better than its predecessors, which mostly focused on either one of these demographic variables. The few that simultaneously accounted for both biomass and density failed to consider their coordinated growth and carrying capacities. However, our model is only valid for established patches where dynamics involve space-filling, not colonization of new areas. Furthermore, its correct estimation of biomass, density and their carrying capacities requires an accurate knowledge of the allometric biomass–density growth. The application of our model demonstrated that seagrass beds at low density have the potential to increase stand biomass rapidly under favorable environmental conditions. Consequently, preventing total loss of meristems is the key to the preservation of seagrass stands, and anchoring shoots at low density in suitable environments should promote rapid restoration. The enhanced knowledge generated by our model is valuable for future research while its enhanced predictive ability is valuable for management efforts.

## Additional files

**Additional file 1.** Matlab executable file running the biomass-density seagrass growth model with parameters calibrated to six seagrass species scattered worldwide.

## Abbreviations

B: biomass
D: density
DW: dry weight
d_algal_: perpendicular distance to the algae IBL
d_grass_: perpendicular distance to the seagrass IBL
IBL: interspecific boundary line
PCA: principal components analysis
RMA: reduced major axis
